# Anlotinib-induced renovascular hypertension and severe coronary artery stenosis: a case report and literature review

**DOI:** 10.3389/fonc.2025.1719861

**Published:** 2026-01-16

**Authors:** Yao Xiao, Kai Liu

**Affiliations:** Department of Cardiology, West China Hospital, Sichuan University, Chengdu, Sichuan, China

**Keywords:** anlotinib, hypothyroidism, hypertension, renal artery stenosis, acute myocardial infarction

## Abstract

Anlotinib is a multi-target tyrosine kinase inhibitor that has been approved in China as a third-line treatment for advanced non-small cell lung cancer. It exerts anti-tumor effects by inhibiting tumor growth and tumor angiogenesis, and its toxicity also arises from this mechanism. Anlotinib can damage vascular endothelium, thereby inducing cardiovascular toxicity, which leads to hypertension, hyperlipidemia, accelerated atherosclerosis, and the initiation of atherosclerotic cardiovascular disease, ultimately resulting in life-threatening cardiovascular events. To the best of our knowledge, this study is the first case report documenting the development of hypertension, hyperlipidemia, hypothyroidism, subsequent discovery of bilateral renal artery stenosis, and acute myocardial infarction in a young patient receiving anlotinib treatment. This showed anlotinib may accelerate systemic large/medium-sized artery atherosclerosis in the context of complex tumor therapy, causing life-threatening cardiovascular events. Besides, it highlights the necessity of regular monitoring of relevant indicators during follow-up for patients undergoing anlotinib therapy, enabling early detection of warning signs and timely intervention.

## Introduction

Anlotinib is an oral small-molecule multi-target tyrosine kinase inhibitor (TKI) that acts on vascular endothelial growth factor receptor (VEGFR), platelet-derived growth factor receptor (PDGFR), fibroblast growth factor receptor (FGFR), and c-Kit to inhibit tumor growth and angiogenesis (1). In China, it is approved for third-line treatment of advanced non-small cell lung cancer and shows potential in other malignancies like medullary thyroid carcinoma. However, it carries safety risks: VEGFR inhibition increases cardiovascular disease risk (like hypertension and heart failure), with hypertension being the most common adverse reaction in trials. Other side effects include hand-foot skin reactions, elevated thyroid-stimulating hormone, and hyperlipidemia (2, 3). Theoretically, anlotinib-induced metabolic disorders may trigger atherosclerotic cardiovascular disease (ASCVD), worsening hypertension and causing severe events. Here, we report a case of anlotinib-induced ASCVD involving bilateral renal artery stenosis, renovascular hypertension, and life-threatening acute myocardial infarction (AMI).

## Case presentation

A 35-year-old male had a left parotid mass resected, pathologically confirmed as parotid acinar cell carcinoma. Postoperatively, he received intensity-modulated radiotherapy (IMRT) and 2 cycles of cisplatin + paclitaxel chemotherapy, achieving stable disease. His medical history included mild renal insufficiency (serum creatinine level: 116 μmol/L, reference range: 68–108 μmol/L), hyperuricemia, and bilateral renal calculi. Thyroid-stimulating hormone (TSH) and blood lipid levels were within the normal range (TSH level: 0.579 mIU/L, reference range: 0.27–4.2 mIU/L; cholesterol level: 4.61 mmol/L, reference range: 2.8–5.17 mmol/L; triglyceride level: 1.48 mmol/L, reference range: 0.29–1.7 mmol/L). He had no history of smoking, coronary heart disease, hypertension, diabetes mellitus, or major surgeries.

Follow-up chest CT showed increased pulmonary nodules. PET-CT and left lung puncture confirmed parotid carcinoma pulmonary metastasis (adenocarcinoma subtype), with no ECG ST-segment changes and normal thyroid function. He received radiotherapy and started to receive anlotinib (12 mg/day, 2-week-on/1-week-off). Follow-up examination after 2 months of anlotinib treatment found elevated blood lipids (cholesterol level: 7.22 mmol/L; triglyceride level: 3.14 mmol/L). Later chest CT showed regressed pulmonary metastases, but he did not undergo regular examinations during subsequent anlotinib treatment.

**Figure 1:**
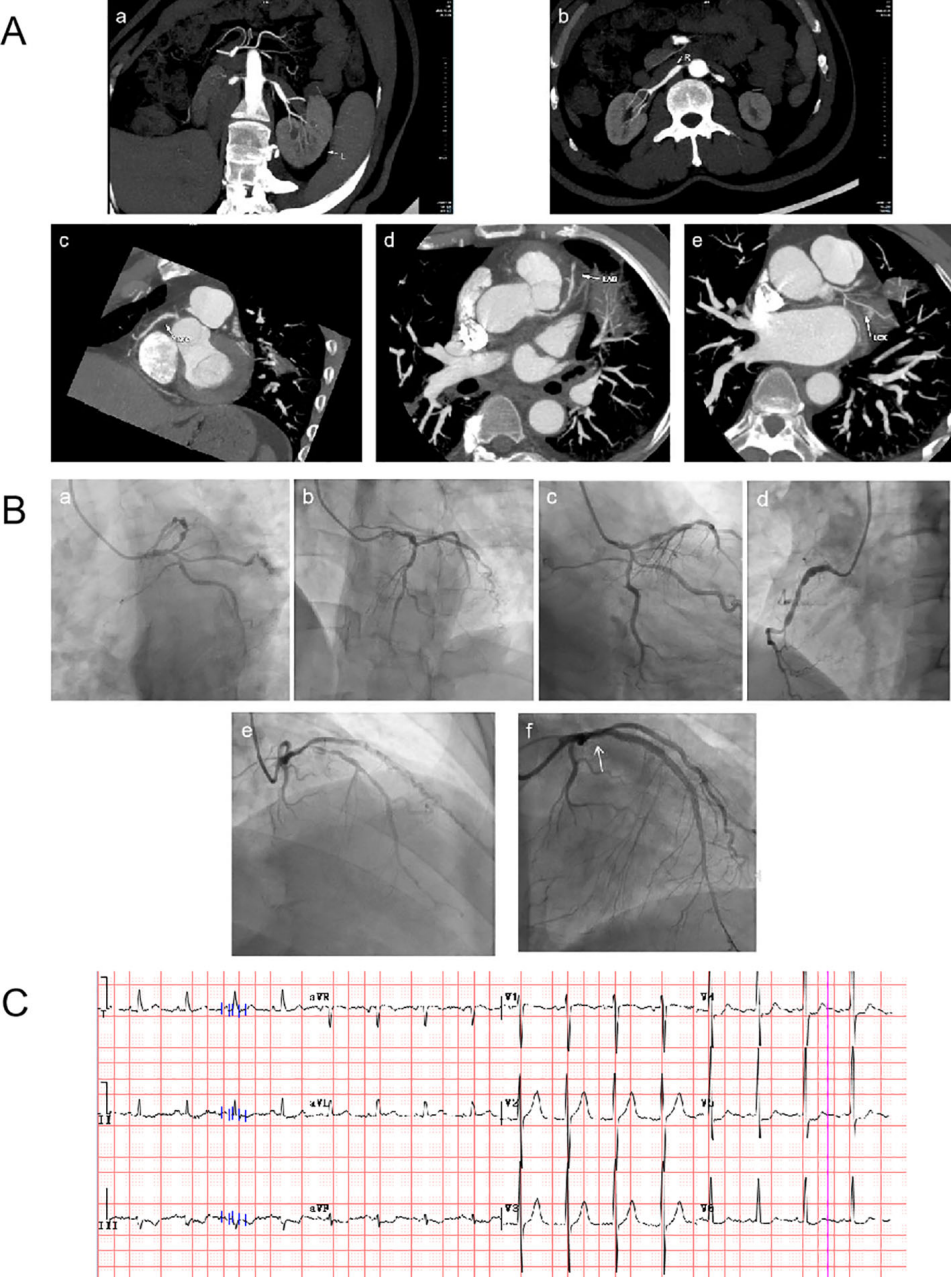
Summary of important images. **(A)** Renal artery and coronary artery computed tomography angiography. (a, b) Severe stenosis at the origin of bilateral renal arteries. (c–e) Severe coronary atherosclerosis with multiple severe stenoses in the proximal RCA and LAD. **(B)** Coronary angiography. (a–e) Moderate left main coronary stenosis, severe LAD/LCX stenosis, and mid-RCA occlusion. (f) Stent placed in the LAD (arrowhead) after emergency percutaneous coronary intervention. **(C)** ECG changes: Extensive ST-segment depression in leads II, III, aVF, and V3-V6. RCA, right coronary artery; LAD, left anterior descending artery; LCX, Left circumflex artery; ECG, electrocardiogram.

The patient was found to have elevated blood pressure, controlled with amlodipine besylate (5mg qd). He later experienced heartburn and chest tightness during strenuous exercise, resolving with rest. His home blood pressure became extremely high that systolic blood pressure exceeded 200 mmHg, and triple antihypertensive drugs including Irbesartan (150 mg qd), prazosin hydrochloride (1mg tid) and amlodipine besylate (5mg bid), failed to control it adequately. Renal vascular CT angiography (CTA) showed severe bilateral renal artery origin stenosis (**Figure 1A**a, b), and he underwent bilateral renal artery balloon angioplasty + stent implantation. The blood pressure decreased after surgery compared with before surgery, but it was still maintained at about 150/100 mmHg. The preoperative and postoperative blood creatinine levels were within the normal range (pre: 88 μmol/L; post: 91 μmol/L).

He was admitted for further hypertension evaluation, receiving diltiazem (90 mg bid) and terazosin (2 mg qn). Examinations revealed mixed hyperlipidemia (cholesterol level: 5.25 mmol/L; triglyceride level: 3.1 mmol/L), hypothyroidism (TSH level: 24mIU/L), average 24-hour blood pressure of 147/103 mmHg, ECG ST-segment changes, and echocardiographic ejection fraction (EF) of 64%. High-sensitivity troponin T and N-terminal pro-B-type natriuretic peptide (NT-proBNP) was tested and no obvious abnormalities were found (high-sensitivity troponin T level: 15.5 ng/L, reference range: < 14 ng/L; NT-proBNP level: 170 ng/L, reference range: < 121 ng/L). No abnormalities were found in other secondary hypertension screening, blood glucose, or renal function. The drug regimen was adjusted to target blood pressure control (nifedipine 30 mg bid + olmesartan 20 mg qd), single antiplatelet therapy (aspirin 100 mg qd), lipid-lowering therapy (atorvastatin 20 mg qn + evolocumab 140 mg q2w), and adjunctive treatment with levothyroxine sodium. Concurrently, targeted therapy was discontinued. However, no significant change in blood pressure was observed compared with the pre-adjustment baseline.

Considering ECG changes and renal artery atherosclerosis, coronary artery computed tomography angiography (CCTA) was performed, that showed severe coronary atherosclerosis(**Figure 1A**c-e). Coronary angiography revealed moderate left main coronary stenosis, severe left anterior descending artery (LAD)/left circumflex artery (LCX) stenosis, and mid-right coronary artery (RCA) occlusion (**Figure 1B**a–e). Post-angiography, he developed contrast-induced acute kidney injury (serum creatinine: 591 μmol/L), with mildly elevated high-sensitivity troponin T (20.7 ng/L) and NT-proBNP (2267 ng/L). Therefore, he received continuous renal replacement therapy (CRRT) via femoral vein catheterization, with evaluation for coronary artery bypass grafting (CABG) indications.

**Figure 2:**
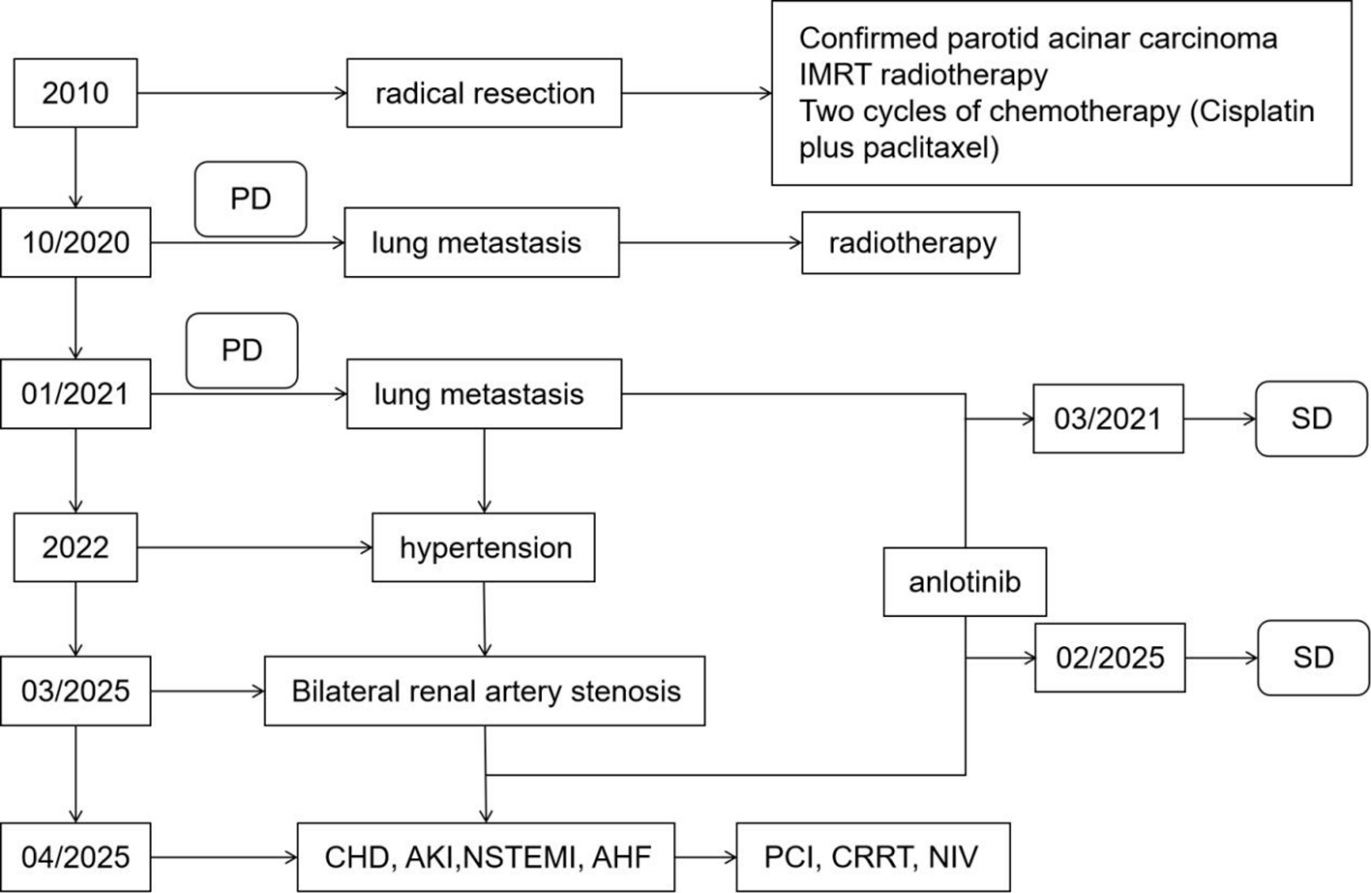
Flowchart of the treatment process. CHD, coronary heart disease; AKI, acute kidney injury; NSTEMI, acute non—ST-elevation myocardial infarction; AHF, acute heart failure; PCI, percutaneous coronary intervention; CRRT, continuous renal replacement therapy; NIV, non-invasive ventilation; PD, progressive disease; SD, stable disease.

Unfortunately, this patient suffered acute non-ST-segment elevation myocardial infarction (NSTEMI) and acute left heart failure while waiting for CABG. Lab tests showed elevated high-sensitivity troponin T (355 ng/L) and NT-proBNP (111359 ng/L), decreased echocardiographic EF (36%), with ECG showing multiple lead ST-segment depression (**Figure 1C**). Emergency percutaneous transluminal coronary angioplasty (PTCA) + stent implantation of the LAD was performed (**Figure 1B**e, f). Follow-up showed declining cardiac markers/NT-proBNP and improved renal function. At discharge, he took arotinolol (10 mg bid) + furosemide (20 mg qd), with blood pressure maintained at 125/87 mmHg (a flowchart of the treatment process is shown in **Figure 2**).

## Discussion

Previous studies reported anlotinib-induced AMI (4). To our knowledge, this is the first case of anlotinib-induced bilateral renal artery stenosis, renovascular hypertension, and AMI, highlighting systemic ASCVD during anlotinib treatment.

The patient suffered from radiotherapy and chemotherapy with paclitaxel/cisplatin before receiving anlotinib. Paclitaxel/cisplatin may increase early ASCVD risk (5), but no significant dyslipidemia or ECG changes occurred within 10 years after chemotherapy. Radiotherapy can cause vascular damage, but radiotherapy-induced AMI is linked to radiation field/dose (6), and the patient’s atherosclerotic lesions involved extensive vessels. So radiotherapy/chemotherapy were ruled out as major cause, while they may have contributed to the patient’s cardiovascular outcomes in a “multiple-hit” manner.

By analyzing the timeline of the patient’s disease progression, mixed hyperlipidemia developed approximately 2 months after the initiation of anlotinib treatment. Hypertension was detected within 1 year of treatment, and angina-like symptoms appeared within 2 years. This suggests that metabolic disorders and systemic vascular changes had occurred in the patient. Furthermore, the patient’s blood pressure decreased significantly compared with preoperative levels after bilateral renal artery stent implantation, although the blood pressure did not return to normal. This still suggests that hypertension was partially caused by renal artery stenosis. Therefore, the subsequent development of multiple atherosclerotic lesions, as well as bilateral renal artery stenosis, hypertension, and AMI, can be associated with anlotinib treatment. In conclusion, while the complex oncological treatment regimen provided the underlying basis for the subsequent major cardiovascular event, anlotinib administration emerged as the critical final triggering factor that precipitated the adverse outcomes.

Potential mechanisms of anlotinib-induced hypertension include altered nitric oxide, endothelin-1, microvascular rarefaction, and selective vasoconstriction (7). As a VEGF signaling inhibitor, anlotinib impairs endothelial nitric oxide synthase (eNOS) via the Akt/PKB pathway. Increased oxidative stress and endothelin system activation may cause eNOS uncoupling (5), reducing nitric oxide synthesis and promoting endothelial damage. Metabolic disorders (hyperlipidemia, hypothyroidism) are common TKI-related adverse events (8). Study proposed anlotinib affects liver cholesterol metabolism via LDL receptor (LDLR)-mediated uptake and AMPK/mTOR pathway (9), accelerating arterial plaque formation and instability. All contributed to ASCVD and poor blood pressure control.

Previous studies reported common anlotinib adverse events including hypertension, hyperlipidemia, hypothyroidism, and hand-foot skin reactions (2, 3). Subsequent studies added cardiotoxic events (shown in **Table 1**) (1, 4, 10–14). Notably, these adverse events are also observed with other TKIs (15). This case shows anlotinib may accelerate systemic large/medium-sized artery atherosclerosis, causing life-threatening cardiovascular events, and suggests alertness for peripheral arterial stenosis in patients with uncontrollable blood pressure on anlotinib.

**Table 1:** Summary of cardiovascular adverse events during anlotinib treatment.

Cardiovascular side effects	Content	References
hypertension	Almost all trials pointed out that hypertension was the most common adverse events of anlotinib treatment, with the highest incidence rate being 67.7% in Han et al’s study.	(10)
myocardial enzymes abnormal	Sun et al. found a 14% incidence of myocardial enzymes abnormal in 21 patients receiving anlotinib at doses of 12 mg/day.	(11)
acute myocardial infarction	The case study by Liu G’s et al. was the first to report on a patient who developed hypertension, hyperlipidemia and angina pectoris, and eventually experienced AMI, following treatment with anlotinib.	(4)
arrhythmia	Shang et al. found new-onset arrhythmias was observed in 39 of 357 (10.92%) patients who received anlotinib monotherapy, including QT prolongation (7.8%), supraventricular arrhythmias (1.4%), conduction disorders (2.0%) and premature contractions (2.0%). Besides, a case report pointed out that sick sinus syndrome was associated with the use of anlotinib in non-small cell lung cancer patients.	(1, 12)
aortic dissection	Jiang et al. presented a case of aortic dissection in a 58-year-old male patient with advanced NSCLC without history of hypertension who received anlotinib as third-line treatment.	(13)
thrombus embolism	Cases of deep vein thrombosis and pulmonary embolism had been reported in cancer patients receiving anlotinib combination treatment.	(14)
hyperlipidemia	Basically all studies have suggested that one of the adverse effects of anlotinib was that it caused an increase in triglycerides or cholesterol, and the incidence of this adverse reaction was not low, and in some studies the incidence even exceeded the rate of hypertension.	(11)
thyroid stimulating hormone elevation	The studies by Han et al. and Huang et al. found that the anlotinib group had a incidence of TSH elevation in about 40%.	(10)

Clinicians are advised to perform baseline risk assessments, conduct continuous monitoring of blood pressure, blood lipid levels and thyroid function, and arrange regular vascular ultrasonography for patients receiving anlotinib therapy, in strict accordance with the guidelines of the 2022 European Society of Cardiology (ESC) on cardio-oncology (16). If warning signs appear, secondary prevention (such as lipid-lowering drugs and antiplatelet agents) should be implemented.

However, this case has limitations: the patient did not undergo regular follow-up during anlotinib treatment, leading to discontinuous biochemical indicator collection, but key data at important time points were intact. Additionally, pre-anlotinib cardiovascular function, renal vascular CTA and coronary angiography were not performed to assess baseline vascular stenosis. Long-term cardiovascular outcomes after anlotinib discontinuation were not assessed (limited follow-up duration), therefore, it remains unclear whether vascular injury is reversible or progressive in the long term. This report could not clarify whether toxicity is related to dosage, duration, or individual susceptibility to anlotinib, which requires further clinical studies to investigate.

## Data Availability

The original contributions presented in the study are included in the article/supplementary material. Further inquiries can be directed to the corresponding author.
